# The data scientist as a mainstay of the tumor board: global implications and opportunities for the global south

**DOI:** 10.3389/fdgth.2025.1535018

**Published:** 2025-02-06

**Authors:** Myles Joshua Toledo Tan, Daniel Andrew Lichlyter, Nicholle Mae Amor Tan Maravilla, Weston John Schrock, Frederic Ivan Leong Ting, Joanna Marie Choa-Go, Kishi Kobe Francisco, Mickael Cavanaugh Byers, Hezerul Abdul Karim, Nouar AlDahoul

**Affiliations:** 1 Department of Electrical and Computer Engineering, Herbert Wertheim College of Engineering, University of Florida, Gainesville, FL, United States; 2 Department of Epidemiology, College of Public Health & Health Professions and College of Medicine, University of Florida, Gainesville, FL, United States; 3 Biology Program, College of Arts and Sciences, University of St. La Salle, Bacolod, Philippines; 4 Department of Natural Sciences, College of Arts and Sciences, University of St. La Salle, Bacolod, Philippines; 5 Department of Chemical Engineering, College of Engineering and Technology, University of St. La Salle, Bacolod, Philippines; 6 Department of Electronics Engineering, College of Engineering and Technology, University of St. La Salle, Bacolod, Philippines; 7 Yo-Vivo Corporation, Bacolod, Philippines; 8 College of Medicine, University of Florida, Gainesville, FL, United States; 9 College of Pharmacy, University of Florida, Gainesville, FL, United States; 10 VA North Florida/South Georgia Veterans Health System, Gainesville, FL, United States; 11 Department of Clinical Sciences, College of Medicine, University of St. La Salle, Bacolod, Philippines; 12 Division of Oncology, Department of Internal Medicine, Corazon Locsin Montelibano Memorial Regional Hospital, Bacolod, Philippines; 13 Department of Internal Medicine, Dr. Pablo O. Torre Memorial Hospital, Bacolod, Philippines; 14 Department of Radiology, The Doctors’ Hospital, Inc., Bacolod, Philippines; 15 Department of Diagnostic Imaging and Radiologic Sciences, Corazon Locsin Montelibano Memorial Regional Hospital, Bacolod, Philippines; 16 Department of Civil and Coastal Engineering, Herbert Wertheim College of Engineering, University of Florida, Gainesville, FL, United States; 17 Faculty of Engineering, Multimedia University, Persiaran Multimedia, Cyberjaya, Malaysia; 18 Department of Computer Science, Division of Science, New York University Abu Dhabi, Abu Dhabi, United Arab Emirates

**Keywords:** cancer care, data science, global south, low- and middle-income countries, medical artificial intelligence, personalized medicine, tumor board, transdisciplinarity

## 1 Introduction

### 1.1 Importance of tumor boards in cancer treatment

Tumor boards are multidisciplinary teams of healthcare professionals that are working together to encompass the full spectrum of care around diagnosing, planning treatment, and advising outcomes for individual cancer patients. These boards typically consist of oncologists, radiologists, pathologists, geneticists, surgeons, nurse practitioners, and other palliative care professionals (1). These boards create a collaborative space for experts from various disciplines to assess clinical factors and patient circumstances, ensuring the application of appropriate care standards and personalized recommendations from the National Comprehensive Cancer Network (NCCN) Guidelines to enhance cancer treatment are met. Since no patient fits the “textbook” cancer profile, oncologists benefit from discussing tailored treatment plans and learning from their colleagues' experiences. When tumor boards are functioning well, they can have a significant impact on patient care (2). For instance, a thoracic oncology board in Munich, Germany, found that 90% of their recommendations met or exceeded clinical standards, with nearly 90% being implemented in practice (3).

Tumor boards are increasingly used worldwide, but expertise and resources for conducting multidisciplinary tumor boards are still limited in the Global South. However, this does not mean they cannot be implemented in developing countries. A 2020 survey from Southeast Asia found that 80.4% of pediatric solid tumor units had pediatric-trained specialists, including oncologists, surgeons, radiologists, pathologists, radiation oncologists, nuclear medicine physicians, and nurses. This indicates that multidisciplinary tumor boards are already in place and that these specialists play a critical role in cancer care (4). With full implementation in the global south, data scientists can further enhance tumor boards with AI and data analytics to improve decision-making and personalize cancer care.

### 1.2 Growing role of data in clinical decision-making

Advances in big data, machine learning (ML), and artificial intelligence (AI) provide more precise, evidence-based, and patient-specific care, thus, giving a different approach as to how healthcare professionals diagnose, treat, and manage their patients (5). For instance, there is a growing number and complexity of data in the healthcare industry such as from Electronic Health Records (EHRs), next-generation genomic sequencing (NGS), and advanced imaging modalities like X-ray Radiography, Magnetic Resonance Imaging (MRI), and Computed Tomography (CT) scans. However, analyzing these data, individually and manually, can be time-consuming and considerably impractical. This is where clinical decision support systems (CDSS) powered by AI and ML are put into action. These systems provide predictive analysis of the disease progression or prognosis, personalized treatment based on the patients' genomic profile, and drug-drug interaction alerts (5, 6). As precision medicine and big data continue to evolve, healthcare will increasingly rely on data-driven tools to enhance patient care, reduce errors, and improve overall health outcomes (7). Data scientists are critical to this process as they can analyze large datasets to identify biomarkers that can predict how a patient will respond to specific treatments (8). In addition, AI algorithms are being used to interpret radiological images, detect early signs of cancer, and predict tumor progression. These tools are increasingly becoming standard in tumor boards, especially in high-income countries (9, 10).

In oncology, the most commonly used diagnostic tools to identify biomarkers and guide targeted therapies in precision medicine are Polymerase Chain Reaction (PCR), fluorescent *in situ* hybridization (FISH), and immunohistochemistry (IHC) to identify biomarkers and guide targeted therapies (11). However, high-throughput next-generation sequencing (NGS)-based diagnostics, which analyze somatic mutations in tumors, have proven clinically useful in identifying single-nucleotide mutations, insertions, deletions, and large genomic rearrangements (12). Thus, multigene NGS testing can provide the oncologist a clinical picture of the patients' molecular profile which can be utilized in planning the best treatment option (13).

### 1.3 Why data scientists should be essential members of tumor boards

As precision medicine continues to gain prominence and the molecular characterization of individual cancers becomes increasingly complex (8, 14), incorporating data scientists into tumor boards is essential. Data scientists bring advanced expertise in ML, data analysis, and bioinformatics, enabling tumor boards to make more accurate, evidence-based clinical decisions that lead to improved patient outcomes (8, 15). They play a critical role in synthesizing and analyzing diverse datasets generated in oncology care, uncovering actionable insights, and informing treatment strategies. This role is particularly crucial as cancer treatment shifts focus toward personalized approaches based on the genetic and molecular characteristics of tumors (16). Specifically, data scientists apply advanced statistical techniques including survival analysis, clustering, and predictive modeling to uncover actionable insights and inform treatment decisions. Their knowledge of foundation models, such as Generative Pre-trained Transformer (GPT), Bidirectional Encoder Representations from Transformers (BERT), and memory-augmented neural networks enables them to extract valuable insights from unstructured data, such as medical records and pathology reports (6, 8).

## 2 Global perspective: the role of data scientists in tumor boards

### 2.1 Overview of global trends in integrating data-driven approaches to oncology

Globally, there is a growing trend towards integrating data-driven approaches into cancer care. In high-income countries, AI-based tools are being used to assist clinicians in interpreting medical images, predicting patient outcomes, and identifying optimal treatment strategies. In the United States, for example, institutions like Memorial Sloan Kettering Cancer Center are using AI tools to guide clinical decisions in oncology (6). These tools, powered by machine learning algorithms, can analyze patient data in real time and provide recommendations during tumor board discussions. However, the integration of these tools varies across regions, with some low- and middle-income countries facing significant barriers to adoption due to a lack of infrastructure and expertise (17).

### 2.2 Examples from high-income countries where data scientists are key members of tumor boards

In high-income countries like the United States and those in Europe, data scientists are already key members of tumor boards. For instance, data scientists at the University of Florida collaborate with oncologists to develop personalized treatment plans based on genomic data and machine learning models (18). These experts apply AI-driven tools to predict patient responses to various therapies and identify potential clinical trials. Similarly, in Germany, data scientists work with oncologists to analyze real-world data and integrate it into clinical decision-making, ensuring that each patient receives the most appropriate care based on their unique profile (19).

### 2.3 Benefits of data-driven insights for personalized cancer treatment

Data-driven insights provided by data scientists have revolutionized personalized cancer treatment. By analyzing large-scale genomic and clinical datasets, data scientists can identify mutations driving cancer and suggest targeted therapies that are likely to be more effective than standard treatments (20). Predictive models can also be used to forecast patient outcomes, allowing oncologists to tailor treatment plans based on the predicted response. These data-driven approaches reduce the trial-and-error nature of cancer treatment and lead to more efficient and effective care (21).

### 2.4 Challenges of integrating data science into medical practice on a global scale

AI is a promising innovation in medical imaging, with applications ranging from image acquisition and processing to reporting, follow-up planning, and data management. Given the broad scope of these applications, AI is anticipated to have a significant impact on the daily work of radiologists (22). The challenge, however, lies in integrating AI-powered equipment due to the lack of information and training provided to many healthcare professionals, especially radiologists. This lack of preparation may contribute to their reluctance to adopt AI in radiology or other healthcare fields (22, 23). Nevertheless, the most significant and transformative advancements in AI are not only occurring in academic hospitals and highly advanced facilities, but in regions and communities grappling with the greatest healthcare challenges and disparities (24).

While high-income countries have made significant strides in integrating data science into oncology care, many LMICs face significant challenges. These include a lack of computational infrastructure, insufficient access to high-quality datasets, and a shortage of trained professionals capable of using AI and machine learning tools (25). Additionally, there are concerns about algorithmic bias and the ethical implications of using AI in healthcare, particularly in countries with diverse patient populations (26). Overcoming these challenges will require significant investment in both technology and human capital, as well as the development of ethical frameworks for the use of AI in clinical settings.

## 3 The case for the global south: maximizing the impact of data scientists

### 3.1 Overview of cancer care challenges in the global south

In the Global South, which includes LMICs or developing regions in Asia, Africa, and Latin America or the Caribbean (27), cancer care is often hampered by a lack of access to advanced diagnostic tools, a shortage of healthcare professionals, and outdated infrastructure. These challenges contribute to higher rates of late-stage cancer diagnoses and poorer outcomes compared to high-income countries (28). In many parts of Sub-Saharan Africa and Southeast Asia, patients often travel long distances to access healthcare, leading to delays in diagnosis and treatment (29). Moreover, many healthcare systems in these regions are underfunded and lack the resources to adopt the latest advances in cancer care. Just like in the Philippines, the main challenge of cancer care is the difficulty of access to global standards of care due to the financial toxicity it brings to the patients and their family (30). Thus despite global and national recommendations to incorporate precision medicine in the care of patients with cancer in the country (31) it is still very challenging for clinicians to incorporate this in their day-to-day clinical practice. If cost were not a limiting factor, oncologists in the Philippines would incorporate AI in precision medicine in managing their patients (32).

### 3.2 Leveraging AI and machine learning: how data scientists can address disparities and improve early diagnosis and treatment

Data scientists have the potential to address healthcare disparities in the Global South by leveraging AI and machine learning to optimize resource allocation and improve diagnostic accuracy. AI-powered tools, such as telemedicine platforms and mobile health (mHealth) applications, can be used to provide real-time diagnostic support in rural areas where access to healthcare professionals is limited (33, 34). ML models can also be used to predict cancer progression, identify patients at high risk of developing complications, and prioritize those most in need of treatment (5). IBM's Watson for Oncology (WFO), an AI CDSS for oncology therapy selection (35), is also found to be a beneficial tool in cancer care in LMICs (17). Hence, these tools can track disease progression and monitor treatment responses, enabling more personalized care (9). By developing predictive models that can be applied even in the absence of advanced diagnostic equipment, data scientists can help improve outcomes for cancer patients in these regions.

While there are many studies discussing the potential of AI in healthcare settings in LMICs, the full implementation of AI in healthcare or specifically in oncology are limited. In Kenya, for instance, AI-driven mobile applications are being used to screen for cervical cancer in rural areas, significantly reducing the time to diagnosis and treatment. While in Ethiopia, machine learning algorithms have been used to analyze blood smear images to diagnose leukemia with high accuracy (34). These examples demonstrate the potential for data science to revolutionize cancer care in the Global South by providing affordable, scalable solutions to some of the most pressing challenges. Moreover, the United States Agency for International Development (USAID) has already been making efforts to address the gap by identifying the challenges to large-scale AI implementation in LMICs and highlighting the actions that can most effectively promote the appropriate use of AI to enhance health outcomes in these settings (36).

## 4 Transdisciplinarity for improved patient care: the future of cancer treatment

### 4.1 Broader transdisciplinarity in healthcare

Transdisciplinarity has emerged as a critical approach in healthcare, particularly in cancer treatment, where multiple fields of expertise are integrated to tackle complex problems from various angles. This collaborative approach incorporates professionals from diverse domains such as medicine, bioinformatics, data science, social sciences, and ethics. By pooling knowledge from these fields, healthcare providers can offer more comprehensive and personalized care for patients, especially in oncology (37). For instance, in the Oncology Complex of Hospital S.G. Bosco in Turin, nurses and other healthcare professionals such as oncologists, psychologists, and social workers have worked together to identify the unmet needs of patients and develop innovative projects to address them (38). Transdisciplinary teams have also been proven to be a successful strategy in expediting emergency department (ED) patient flow. Through the collaboration of nurses and other allied health professionals, the team was able to address patients' needs more efficiently, ensuring prompt delivery of care (39). Additionally, a secondary analysis of the International BRIGHT Study on chronic illness management after heart transplant revealed that centers with dedicated multidisciplinary teams achieved better outcomes (*p* = 0.042) (40). Similar examples are increasingly found across medical disciplines, including efforts to leverage data resources, such as a project linking 54 million electronic health records in England (41). Thus, these efforts highlight the potential for data scientists to make a significant impact within transdisciplinary teams across various healthcare sectors.

### 4.2 Qualifications and competencies for tumor board data scientists

To effectively contribute to tumor boards, data scientists must meet a range of technical, domain-specific, and interpersonal requirements. A solid foundation in data analysis, statistical modeling, and machine learning is essential, particularly with expertise in oncology-related datasets such as omic data, medical imaging, and electronic health records. Candidates should hold a graduate-level degree in a discipline with a strong emphasis on statistics and data science, such as mathematics, statistics, physics, computational biology, computer science, electrical engineering, biomedical engineering (BME), or related fields. This level of expertise ensures the ability to handle large and heterogeneous datasets while adhering to healthcare regulations similar to the Health Insurance Portability and Accountability Act (HIPAA) of the United States to maintain patient data privacy and confidentiality.

A robust understanding of medical terminology and clinical workflows enables seamless communication with oncologists, radiologists, pathologists, and other healthcare professionals. Furthermore, data scientists must excel in translating complex findings into actionable insights, employing data visualization techniques that facilitate understanding across diverse disciplines. Beyond technical skills, strong collaboration and communication abilities are vital for integrating effectively into the multidisciplinary environment of tumor boards. To ensure the quality and consistency of their contributions, eligibility for this role may require regulation by national or international professional bodies. Lastly, a commitment to continuous learning in both data science and oncology ensures that data scientists can adapt to emerging challenges and innovations in precision medicine.

Insights from the study of Fermin and Tan (42), on the development of BME as a formal discipline, particularly in some LMICs in the Global South, highlight the critical importance of formalized educational pathways and professional recognition in integrating technical expertise into healthcare. This research has demonstrated that LMICs recognizing BME as an academic and professional field have significantly advanced healthcare outcomes, leveraging limited resources to achieve impactful innovations. Applying these lessons to the integration of data scientists in tumor boards emphasizes the need for structured education programs and national or international regulatory frameworks tailored to LMIC contexts.

Efforts to standardize the qualifications and competencies of data scientists include frameworks such as the EDISON Data Science Framework (EDSF), which provides a comprehensive foundation for the professionalization of data science, comprising components such as the Competence Framework (CF-DS), the Body of Knowledge (DS-BoK), the Model Curriculum (MC-DS), and the Professional Profiles (DSPP) (43–46). In the United States, the American Medical Informatics Association (AMIA) emphasizes competency-based accreditation for health informatics, which aligns closely with data science roles (47). The Association for Computing Machinery (ACM) further supports this with computing competencies for undergraduate data science curricula, detailing essential knowledge and skills (48).

Country-specific regulations vary; for example, the United States emphasizes skills-based hiring under Executive Order 14110 for AI and data professionals, with a focus on practical competencies over formal education (49). Similarly, the National Occupational Standards (NOS) of the United Kingdom include a standard which outlines detailed performance criteria for computational data analysis in life sciences [Unique Registration Number (URN) COGBIO-05], making it particularly applicable to specialized data science roles (50).

### 4.3 Key tasks of a data scientist in tumor boards

The data scientist plays a vital role as a synthesizer of knowledge, integrating patterns from large, disparate datasets across multiple domains, including clinical, genomic, and environmental data (51). Within tumor boards, data scientists do not only leverage advanced ML models but also employ a variety of statistical and computational techniques to optimize treatment planning and personalize patient care (8). The breadth of their contribution extends beyond predictive modeling and data integration into more specialized areas such as reinforcement learning, Bayesian networks, simulation-based approaches, and many others. Table 1 summarizes the key tasks for data scientists in tumor boards, the challenges that they face, and data science solutions to overcome them.

**Table 1:** A summary of key tasks for data scientists in tumor boards, the challenges they face, and data science solutions to overcome them.

Tasks, challenges, and solutions for data scientists in tumor boards			
Key task	Example	Challenges	Potential solutions
Reinforcement Learning (RL)	Developing adaptive chemotherapy dosages that minimize toxicity while maximizing effectiveness (55)	Real world oncology environments are non-Markovian, and assumptions that they are may lead to misrepresentation of clinical scenarios (54)	Employ partially observable Markov decision processes or multiple imputation methods to improve the environment representation (54)
Bayesian networks	Guiding immunotherapy decisions for head and neck cancer by integrating patient data and treatment probabilities (57)	Handling incomplete clinical histories and subjective data in Bayesian Network construction often leads to inaccurate probabilistic inferences (57)	Integrate expert knowledge with advanced imputation methods to refine Bayesian Network structures and enhance predictive accuracy (57)
Simulation-based approaches	Optimizing Tumor Treating Fields (TTFields) for brain tumors by simulating array placement and tumor characteristics (59)	Simulation-based approaches often struggle with the high computational demands required for accurate and timely results in clinical settings	Partnerships between national governments and hardware providers such as Nvidia to develop supercomputer-level capabilities
Dose optimization and scheduling	Recent DL models predict doses by analyzing patient scans to map optimal dose values, guiding treatment planning and predicting dose distributions (62)	AI-driven dose prediction struggles with generalizability across institutions due to variations in patient populations and treatment practices, including treatment planning	Develop collaborative multi-center databases to train models on diverse datasets for better generalization. Incorporate domain adaptation techniques and robust cross-validation strategies to refine model performance across varying clinical settings
Multi-omics integration	Selecting appropriate methods to maximize accuracy in cancer classification, drug response prediction, and runtime efficiency (65)	Discovering true causal relationships among genomic, transcriptomic, and proteomic layers is challenging due to high-dimensional data and batch effects from inconsistent datasets generated across multiple sites	Apply causal modeling with Bayesian networks and biological knowledge to improve integration accuracy, using batch effect correction and robust normalization for consistent, reliable data
Radiomics and pathomics integration	Developing models to classify cancer stages or predict survival outcomes by integrating radiological and pathological imaging data (66)	Radiopathomics currently struggles with data standardization, feature dimensionality reduction, and model generalizability across diverse patient populations	Use advanced feature selection methods to reduce feature redundancy and improve model robustness. Incorporate multi-center data and external validation cohorts to ensure model applicability across different clinical settings
Advancing cancer care through spatial biology	Using spTx to understand breast cancer drug sensitivity across tumor regions, improving treatment personalization (73)	Scaling data integration, handling computational demands, and translating complex spatial insights into actionable therapeutic strategies	Develop streamlined workflows that employ cloud-based visualization techniques to translate spatial insights into clinically relevant therapeutic decisions
Causal modeling in oncology	Applying a large language model to determine the directionality of causation in NSCLC, such as how smoking status may impact treatment choice (76)	Causal discovery in oncology datasets is hindered by the complexity of accurately identifying causal relationships in multi-modal data, e.g., genomics and electronic health records, and validating generated causal structures	Combine causal discovery algorithms with domain expertise encoded through LLMs to refine causal graphs. Validation techniques, such as Bayesian Dirichlet scoring, can ensure the causal models align with both observed data and clinical relevance
Adaptive treatment strategies	Modifying radiotherapy plans during treatment to account for tumor changes and improve precision (79)	Limited rapid imaging, precise segmentation, and real-time replanning capabilities for MRI-guided adaptive radiotherapy and automated planning tools	Fund research which may advance accelerated imaging, deep learning auto-segmentation, and real-time adaptive planning capabilities
Decision support systems	A CDSS based on ML that integrates clinical, imaging, biologic, and genetic data to aid tumor board discussions of metastatic lung cancer cases (82)	Lack of precise and standardized data on tumor locations, sizes, and numbers in clinical notes can lead to limitations in training ML models for CDSS	Extract geometric features from medical images, like tumor volume and location, to enhance ML models for precise treatment predictions
Optimization of combination therapies	Designing nanoplatforms for synergistic drug delivery and reduced toxicity in cancer treatment (84)	Variability in the TME and immune profiles makes it difficult to design therapies that effectively modulate immune responses across diverse patient populations	Use causal inference models to integrate genetic, proteomic, and spTx data for identification of key biomarkers and pathways to design personalized combination therapies

#### 4.3.1 Reinforcement learning (RL)

Reinforcement learning, a type of machine learning where an algorithm learns to make sequences of decisions by maximizing cumulative rewards, has growing applications in treatment planning (52). Data scientists need to account for the differences between the oncology environment and a classic RL environment following the Markov assumption that the future state of a system depends only on its current state (53). They may do this by suggesting an adaptation to a RL model. In oncology, RL is used to develop dynamic treatment strategies that adapt based on the patient's response to ongoing treatments. RL algorithms can continuously adjust dosages of radiation or chemotherapy to minimize toxicity while maximizing effectiveness (54). Moreover, Tempo, a novel framework based on RL for personalized screening, has proven its effectiveness in the context of breast cancer. The Tempo policy, combined with an AI risk model, outperforms current practices in early detection and can be adapted to different screening preferences. It also improves detection while reducing overscreening (55). A personalized treatment approach allows clinicians to adjust treatment plans in real time, providing adaptive strategies as new patient data emerges. Similarly, personalized screening tailors assessments to individual risk profiles, enhancing early detection and treatment precision.

#### 4.3.2 Bayesian networks

Data scientists also use Bayesian networks, probabilistic graphical models that represent a set of variables and their conditional dependencies. These networks help in predicting outcomes and understanding the likelihood of various treatment responses based on observed data. In a tumor board setting, Bayesian networks can integrate information from different sources—clinical data, molecular biomarkers, and patient history—to estimate the probabilities of different clinical outcomes and provide uncertainty quantifications, helping doctors make more informed decisions in cases where there is ambiguity (56). For example, in the study of Huehn et al. (57), they developed a digital patient model using Bayesian networks to integrate patient data and relevant probabilities for treatment decisions in head and neck squamous cell carcinoma (HNSCC). Validation showed the model effectively guides immunotherapy decisions, with 84% accuracy and significant concordance (Cohen's *κ* = 0.505, *p* = 0.009) when compared to actual treatment decisions for 25 patients. This model was created to represent a physician's decision-making in tumor boards, integrating clinical and molecular data to identify the best treatment for each patient.

#### 4.3.3 Simulation-based treatment planning

Simulation-based approaches enable data scientists to create virtual treatment scenarios and evaluate potential outcomes. By simulating multiple strategies, clinicians can explore the consequences of various plans before applying them to patients. These simulations are especially valuable in complex cases with multiple options, helping model long-term effects of treatments like chemotherapy combined with immunotherapy or predict patient responses based on genetic profiles (58). Just like in the study of Federov et al. (59), they developed a novel simulation-based method for optimizing Tumor Treating Fields (TTFields) treatment planning for brain tumors. TTFields, delivered through transducer arrays on the skin, inhibit tumor growth, with their distribution varying based on array placement, patient anatomy, and tumor characteristics. Incorporating such algorithms into advanced treatment planning systems is expected to enhance physician management of TTFields patients, ultimately improving patient outcomes.

#### 4.3.4 Dose optimization and scheduling

In addition to simulating treatment outcomes, data scientists also use dose optimization models to personalize the amount and timing of therapies such as chemotherapy or radiation. These models aim to balance efficacy with minimizing side effects, especially in treatments that involve toxic agents. By adjusting dosing schedules based on individual patient metabolism and tumor characteristics, clinicians can optimize therapy duration and frequency to increase the probability of success without compromising patient quality of life (60). The emergence of new anti-tumor treatments further complicates this, creating an urgent need for optimized dose-schedule strategies. Data scientists can be useful by using Bayesian adaptive designs which can offer an efficient approach to assess multiple doses and schedules concurrently within a single clinical trial (61). Moreover, recent advancements in deep learning (DL) have led to the development of DL-based dose prediction models. Unlike traditional methods, DL automatically extracts features from patient CT, MRI, or PET scans to map optimal dose values, guiding treatment planning systems for final dose distribution. It can also predict dose distributions based on anatomical data and dose prescriptions (62).

#### 4.3.5 Multi-omics integration

Multi-omics integration is another important facet of treatment planning. Data scientists combine data from genomics, transcriptomics, proteomics, and metabolomics to create a comprehensive molecular profile of a patient's tumor. By integrating these diverse datasets, data scientists help tumor boards develop more targeted therapies. For example, a multi-omics approach might reveal not just a single genetic mutation driving the cancer but also how it interacts with other molecular pathways, leading to more tailored treatments, such as targeting metabolic pathways in addition to the genomic aberration (63). Multi-omics offers a comprehensive view of cancer biology, but the large data volumes pose analytical challenges. AI helps address these by identifying patterns and extracting insights from complex omics data, advancing cancer research (64). Similar to the study by Cai et al. (65), which explored machine learning methods for integrating multi-omics data in cancer research, their focus was on both general-purpose and task-specific approaches. They benchmarked five methods using data from the Cancer Cell Line Encyclopedia, assessing accuracy in cancer classification, drug response prediction, and runtime efficiency. Their paper provides recommendations for selecting appropriate methods and encourages the development of new tools to advance drug discovery and personalized treatments.

#### 4.3.6 Radiomics and pathomics integration

Radiomics involves extracting quantitative features from medical imaging (e.g., CT, MRI, PET scans) that can reveal tumor heterogeneity and predict patient outcomes. Similarly, pathomics analyzes histopathological images to identify patterns that may not be discernible by a pathologist alone. Data scientists combine these image-derived features with clinical and genomic data to improve treatment predictions and select optimal therapeutic strategies. Like in the study of Tan et al. (66), they developed radiopathomics models, employing ML algorithms, that can highly classify stage I, II, and III gastric cancer. Other researchers also developed radiopathomics models to predict prognosis in patients with colorectal and lung cancers, as well as their overall survival (67, 68). Radiomics and pathomics are particularly valuable in situations where genetic testing may not be available, offering non-invasive options for assessing tumor characteristics (69, 70).

#### 4.3.7 Advancing cancer care through spatial biology

Spatial biology technologies, such as GeoMx® (NanoString Technologies®)^1^, CosMx™ (NanoString Technologies®)^2^, Visium® (10x Genomics®)^3^, and Xenium™ (10x Genomics®)^4^, are revolutionizing cancer research by integrating molecular profiling with spatial context. These approaches enable the mapping of tumor heterogeneity, microenvironment, and cell-cell interactions, providing insights that traditional bulk sequencing cannot offer. For instance, spatial transcriptomics (spTx) platforms like CosMx™^5^ and Xenium™^6^ enhance tumor analysis by combining high-resolution imaging with RNA profiling, capturing cellular organization and biomarker localization within tissue samples (71). Visium® HD^7^ offers sub-cellular resolution, enabling more detailed reconstruction of cell morphology and expression patterns in tumors (72).

One of the most notable applications of spatial biology in the field of oncology is the use of spTx in understanding breast cancer. Despite the improved patient outcomes in precision medicine, breast cancer treatment still faces challenges due to intratumoral heterogeneity (ITH), where different parts of the tumour respond differently to drugs. Using spTx, recent research shows that drug sensitivity varies across the tumor, from the core to the periphery. It also finds that genetically identical tumor cells can respond differently depending on their location (73). Thus, it is crucial to consider not only the genetic profile of the tumor but also its spatial organization and the surrounding microenvironment. This approach could lead to more personalized and effective treatments by addressing the tumor's complexity, improving outcomes, and reducing the chances of treatment failure.

#### 4.3.8 Causal modeling in oncology

Causal modeling focuses on determining cause-and-effect relationships, going beyond correlation to uncover how specific interventions lead to observed outcomes. This is particularly critical in oncology, where data scientists use advanced techniques like the Peter-Clark (PC) algorithm (74) and latent Gaussian models to identify causal pathways in treatment effects and disease progression (65). While frameworks like the SHapley Additive exPlanations (SHAP) and Local Interpretable Model Agnostic Explanation (LIME) help interpret machine learning models, they primarily explain correlations rather than causation (75).

For example, a large language model (LLM) was used to improve causal discovery of the factors impacting survival in Non Small Cell Lung Cancer (NSCLC), revealing potentially unexpected causal relationships such as smoking status having a causal effect on treatment choice (76). The role of data scientists extends further, applying causal inference methods, such as inverse probability weighting and structural failure time models, to address biases like treatment switching in survival analysis (77). By integrating these causal approaches into clinical workflows, data scientists enable tumor boards to base decisions on robust cause-and-effect insights, driving better patient outcomes in personalized oncology care.

#### 4.3.9 Adaptive treatment strategies

Another application where data scientists play a key role is in developing adaptive treatment strategies. These strategies are designed to evolve in response to the patient's changing condition. For instance, if a patient's tumor becomes resistant to chemotherapy, data scientists can employ adaptive models to recommend alternative treatments, such as switching to targeted therapy or immunotherapy. By continuously analyzing patient data throughout the treatment course, adaptive models ensure that treatment plans remain flexible and responsive to real-time changes in disease progression (78). For example, adaptive radiotherapy (ART) is used as a cancer treatment method that adjusts the radiation plan during treatment to account for changes in the tumor's size, shape, and position, as well as nearby healthy tissues. This helps make the treatment more effective and safer by targeting the tumor better while reducing harm to healthy tissues, thus improving both the treatment's efficacy and safety (79).

#### 4.3.10 Decision support systems (DSS)

Data scientists also develop decision support systems (DSS) that aid oncologists in selecting treatment pathways based on the integration of patient data, clinical guidelines, and outcomes from past treatments. These systems use a combination of machine learning algorithms and clinical knowledge to provide recommendations that consider both standard care protocols and personalized factors. In particular, data scientists design systems capable of providing real-time decision support by processing data from sensors, wearables, or EHR systems. For example, a clinical DSS could alert the physician in real time about the patient's deteriorating condition, helping to prevent adverse effects or complications (80). Monitoring biometric data in cancer patients offers crucial insights for oncologists. Initial metrics such as steps and heart rate aid in prognosis and treatment choices, while ongoing tracking helps identify potential adverse events and supports rehabilitation monitoring (81). Additionally, a recent thesis from the University of Toronto found that Machine Learning, especially random forests (RF), could be used to create a better clinical DSS (CDSS) for metastatic lung cancer than current hospital decision support framework (82). A one-step prediction of non-local therapy, surgery, or stereotactic body radiation therapy using RF achieved an Area under the Receiver Operator Curve of 0.857 and a mean accuracy of 72.55% against the tumor board's final decision as truth.

#### 4.3.11 Optimization of combination therapies

Data scientists can also help optimize combination therapies, where multiple treatments such as chemotherapy, radiotherapy, and immunotherapy are used in tandem. By modeling the interaction effects between various treatments, they can predict the synergistic or antagonistic outcomes of combining therapies. This is particularly important in oncology, where combination treatments are often necessary but must be carefully balanced to avoid excessive toxicity while maximizing therapeutic benefit (83). For instance, nanoplatforms are used to improve treatment precision and reduce toxicity by enhancing drug delivery, especially in combination with immunotherapy, chemotherapy, radiotherapy, and other cancer treatment procedures (84). With the help of data scientists, AI-powered predictive modeling can be utilized to help simulate intricate nanoscale processes, assisting in the design and refinement of nanomaterials and devices. This technique will enhance the development of nanoplatforms, optimizing their performance in drug delivery and cancer therapy while reducing associated risks.

### 4.4 The importance of foundation models in tumor boards

Foundation models, particularly transformer-based architectures like GPT, BERT, Pathways Language Model (PaLM), and Large Language Model Architecture (LLaMA) (85–88), have significantly enhanced natural language processing (NLP) and pattern recognition in healthcare, making them indispensable tools for tumor boards. These models are adept at processing unstructured clinical data, including medical histories, radiology reports, and pathology images, which often contain critical information for decision-making. By efficiently summarizing and analyzing vast amounts of data, foundation models enable clinicians to make more precise, data-driven decisions that improve patient outcomes (89, 90).

#### 4.4.1 Transformer-based models

These models, including GPT and BERT (85, 86), excel at processing large amounts of textual data from patient records and clinical notes. Their ability to handle unstructured data and perform complex tasks like summarization and sentiment analysis offers significant advantages over traditional statistical methods, which often struggle with the high dimensionality and noise inherent in medical data (91). In tumor boards, transformer-based models can summarize complex patient histories and predict treatment responses, providing oncologists with actionable insights.

#### 4.4.2 Memory-augmented neural networks (MANNs)

Memory-Augmented Neural Networks (MANNs) are designed to enhance long-term dependencies, allowing for the retention of information over extended periods (92). This capability is particularly valuable for tracking the progression of diseases like cancer, where long-term data about tumor growth and treatment responses can inform future care strategies. MANNs are essential in long-term treatment planning, helping clinicians assess how a patient's condition might evolve over time (93).

#### 4.4.3 Graph neural networks (GNNs)

Graph Neural Networks (GNNs) play a crucial role in understanding relationships between biological factors such as gene expressions, protein-protein interactions, and TMEs (94). These models analyze complex biological networks to provide insights into molecular mechanisms that drive cancer progression. By mapping interactions within biological pathways, GNNs help identify potential therapeutic targets, making them valuable for personalized treatment strategies in oncology (95).

#### 4.4.4 Neural ordinary differential equations (neural ODEs)

Neural Ordinary Differential Equations (ODEs) offer a powerful approach to modeling continuous biological processes (96). These models are adept at simulating how a patient's condition changes over time, making them useful for predicting treatment responses and disease progression. Neural ODEs are particularly valuable in dynamic environments like oncology, where patient conditions can rapidly change due to treatment or disease relapse (97).

#### 4.4.5 Hyena hierarchy

The Hyena Hierarchy model is a relatively new advancement in sequence modeling, designed to outperform traditional transformer models in processing long-range dependencies without the quadratic scaling issues inherent in transformers (98). In tumor boards, Hyena Hierarchy can be applied to analyze extensive patient datasets, such as multi-modal data from clinical trials or longitudinal studies. This model's ability to handle long sequences efficiently makes it ideal for extracting patterns from extensive patient histories, genetic sequences, and radiological imaging, leading to more personalized and precise treatment plans.

#### 4.4.6 Fourier transform-based models (FNet)

FNet models replace the self-attention mechanism found in transformers with Fourier Transforms, which are computationally efficient and scalable. FNet models are particularly useful in situations where quick and efficient data processing is required, such as analyzing large datasets of patient vitals or sensor data from wearable devices (99). In tumor boards, FNet could be used to extract meaningful insights from continuous data streams like monitoring patient vitals in real time, offering the potential for more dynamic, real-time decision-making during cancer treatment.

#### 4.4.7 Retentive networks

Retentive Networks are designed to process long-range data while retaining context across large sequences, much like transformer models but with more efficient memory use (100). This model can be useful in oncology, where a continuous stream of patient data is analyzed over the long term. Retentive Networks can help tumor boards track the effectiveness of treatments over time, analyzing data from repeated scans, lab results, and genomic updates to ensure that treatments remain optimal as the disease evolves.

#### 4.4.8 Adaptive computation time models

Adaptive Computation Time is a strategy in DL models that improves computation efficiency and adaptability (101). It is particularly relevant in scenarios where different parts of a dataset require varying amounts of computational attention. In tumor boards, these models can dynamically allocate resources to the most critical patient data, such as prioritizing genomic markers or radiology results in patients with advanced cancers. This ensures that the model focuses on the most relevant data points to assist with urgent clinical decisions.

#### 4.4.9 Autoencoder-based models

Variational Autoencoders (VAEs) play an important role in modeling high-dimensional data, which is common in oncology due to the complexity of genetic and clinical information (102). VAEs can be used to compress these large datasets while retaining the most relevant features for tumor classification or risk stratification. In tumor boards, VAEs assist by reducing data complexity, enabling clinicians to focus on the most critical elements for personalized treatment planning (103).

### 4.5 How these models compare to traditional biostatistics

Traditional biostatistics relies on predefined assumptions, which often limit its capacity to analyze complex and high-dimensional data. While biostatistics has been invaluable for hypothesis-driven research, it may fall short in addressing the complexity of cancer data, especially as datasets grow in size and complexity (104). Machine learning models, particularly foundation models, offer a more adaptive approach, as they can learn from data and adjust predictions in real-time. These models are not bound by the rigid assumptions of traditional biostatistics and are better equipped to handle the evolving nature of cancer treatment paradigms (105).

By working alongside traditional biostatistical methods, data scientists using advanced ML models bridge the gap between established statistical techniques and novel data-driven approaches. This collaboration ensures that tumor boards can extract the most comprehensive insights, allowing for more accurate and personalized cancer care (106).

### 4.6 Expanding roles for improved patient care

Theories around transdisciplinary integration suggest that expanding the roles of healthcare teams to include more diverse professionals—such as data scientists, AI specialists, and social scientists—leads to improved patient outcomes. As healthcare becomes more data-centric, the roles of data scientists are expected to grow even further (16). Frameworks like the learning health system (LHS) propose that constant learning and data integration lead to better clinical outcomes, which aligns with the data scientist's role in synthesizing diverse sources of information (107). Healthcare professionals are also encouraged to embrace and integrate AI in their respective fields to ensure the effectiveness and success of transdisciplinary teams. As essential members of the healthcare team, nurses, for instance, should receive ongoing education and training in AI to prepare for the AI-driven future of healthcare. This can be most impactful when AI concepts and skills are incorporated into the nursing curriculum, ensuring nurses are equipped with the knowledge and tools needed to navigate and leverage AI in their practice (108). Additionally, AI can significantly support radiologic technologists by enhancing their ability to provide high-quality, efficient, and safe imaging services, while also allowing for better collaboration with other healthcare professionals. By automating repetitive tasks and providing decision support, AI helps radiologic technologists focus on more complex aspects of patient care and diagnostics (109).

Healthcare teams that embrace transdisciplinary approaches are better equipped to tackle complex problems, as they benefit from the combined expertise of professionals from different domains. This collaborative approach improves the ability to adapt to new treatments, technologies, and patient needs (110).

### 4.7 Why a data scientist who stays current with foundation models is invaluable

The field of foundation models is rapidly evolving, and most clinicians are likely unable to stay current on these developments in addition to their intense workload. Data scientists who are familiar with the latest models—such as GPT, BERT, PaLM, and GNNs—can integrate insights from clinical, genomic, and lifestyle datasets to inform the tumor board’s decisions. This capability allows tumor boards to remain at the cutting edge of cancer treatment (90).

In resource-constrained settings, such as those in the Global South, a data scientist's ability to leverage foundation models to extract meaningful insights from limited data can be transformative. By applying these models to real-world data, data scientists can improve diagnostic accuracy, optimize treatment plans, and make personalized cancer care more accessible to underserved populations (34).

## 5 Overcoming barriers to data science integration in the global south

### 5.1 Identifying key barriers

The Global South faces several significant barriers to the integration of data science in healthcare, particularly in oncology. Key challenges include:

#### 5.1.1 Limited supercomputer infrastructure in the global south

Here is a brief breakdown of supercomputer power distribution in the Global South by region. Computing power is often quantified by “flops”, where one flop indicates the ability to perform one floating point operation per second. For reference, the values below can be compared to the most powerful supercomputers in the world such as *El Capitan*^8^ in the United States with 1,742 petaflops (1.74e + 18 flops) based on the Top500 supercomputer list in 2024. Unfortunately, there is not a quality report comparing AI task processing speeds which can differ significantly from traditional metrics. For example, the Aurora system^9^ which ranks number three on the Top500 list may be the world's fastest computer for AI tasks. And those computers which could be used for AI tasks may be used for predictive modeling of phenomena such as weather instead. Because of this, some of the descriptions below may over- or under-estimate the systems' usefulness for integrating AI in tumor boards. Also of note, it is believed that China does not publish complete supercomputer specifications for all its systems to public sources.

- **South America** is home to 10 notable supercomputers, the largest of which is the Pégaso^10^ cluster owned by Brazil oil giant Petrobras^11^ with 19 petaflops, or 1.9e + 16 flops. This company owns six out of the ten supercomputers on the continent and mostly uses them for tasks such as geophysical exploration. Brazil is rapidly developing AI infrastructure, but the rest of South America lags behind partly due to political instability.
- On the **African** continent, only Morocco hosts a supercomputer of competitive power, Toubkal^12^, with 3.15 petaflops. Africa is far behind in developing this infrastructure, but countries which are making headway include Morocco, South Africa, Nigeria, and Angolo, among others. These countries can pave the way to fast track the broader continent to a fair and equitable AI revolution.
- **Southeast Asia** is strategically located between regions of AI progress including South Asia, East Asia including China, South Korea, and Japan, Australia, and some connections with the United States. Singapore is currently the clear leader in developing high performance computing infrastructure in the region, especially with the recent launch of the ASPIRE 2A+^13^ with 20 petaflops of processing power and a recent announcement of $270 million in additional investment. While other countries in the region are highly promising for the development of such resources, including Thailand, Indonesia, and the Philippines, many new projects will be needed to realize this potential.

#### 5.1.2 Resource constraints

- **Limited Education and Training**: There is a shortage of data scientists and healthcare professionals trained in advanced data analytics in LMICs. Many healthcare workers lack the skills necessary to operate complex AI systems, hindering the adoption of data science in clinical practice (17).
- **Insufficient Investment in Health Data Systems**: Health systems in LMICs often suffer from underfunding, with limited investment in digital health infrastructure. This lack of financial support prevents the development of integrated health data systems that could facilitate the use of AI and ML in cancer care (111).

#### 5.1.3 Ethical considerations

- **Trust and Privacy of AI Tools**: Trust and privacy have been identified as barriers to using AI to improve healthcare in LMICs. Specifically, some of the centers attempting to use AI have been concerned with transparency and security, such as when using x-rays to diagnose Tuberculosis (112, 113).
- **Combatting Exploitation and Unintended Consequences**: While AI has the potential to improve healthcare in low-resource communities and is championed as a means for global health justice (114, 115), data mobilization in LMICs also holds the potential to exacerbate historical exploitation and increase inequalities. For example, the data wealth disparity between developed and developing countries may contribute to a data-centric neocolonialism in which centers of influence collect data from LMICs without payment or informed consent and use them to develop products which are then sold to LMICs (116, 117). Additionally, AI tools could be used to further ostracise certain people groups within a society such as ethnic minorities, the incarcerated, and women. Yu and Zhai suggest a concept called the “AI Deployment Paradox” to describe this risk and recommend flexible and tight regulation and persistent engagement with local stakeholders to address it.

#### 5.1.4 Risks of over-reliance on AI

A major issue with the power of AI is that practitioners may become overly reliant on it, leading to automation bias. This can result in harmful outcomes like incorrect decisions, overdiagnosis, overtreatment, and defensive medicine (118). These issues arise because AI systems, despite being advanced, can still make mistakes or miss important nuances in a patient's condition. And so, the clinician's supervision is crucial for ensuring the best possible care. On the other hand, patients may also distrust AI and most of them are expressing discomfort with physicians who are solely relying on AI for medical care. This is mainly influenced by factors such as education, knowledge, and experiences on AI. For example, in pain management or surgical procedures, some patients are skeptical about using AI because they believe that it will only worsen their condition or offer no significant benefits. They are also reluctant in using AI chatbots for mental health support without a therapist's involvement because they believe that AI does not offer any emotional support. However, those people with higher education and more experiences in using AI generally view AI as a helpful tool for improving patient care. Thus, skepticisms may have arisen from limited understanding and data privacy concerns (119).

In addition, patients are more than just their health—they have unique needs, wishes, and values. Healthcare is built on a special relationship between doctors and patients, focused on respect, privacy, fairness, and care. This relationship should support patients' choices and protect them from harm. Thus, medical AI should align with these principles (118). Many medical schools present a fictional story about a woman who visits her doctor to receive her biopsy results. Turns out, she has metastatic cancer and was told she has only a few months to live. While the diagnosis and prognosis are precise, backed by copious evidence, it was revealed that the “doctor” delivering the news is actually an AI program with little emotional engagement. Although most oncologists do not fear being replaced by AI, some may be concerned that companies and healthcare professionals might fully rely on AI in the name of efficiency or profit. Thus, data scientists should be aware of the role they might play in this. As data scientists may become more involved in tumor boards, they should emphasize how AI can enhance the patient experience by ensuring they *feel* genuinely cared for.

#### 5.1.5 Professional culture gaps between oncologists and data scientists

- **Epistemic and Communication Gaps:** Data scientists and clinicians have distinct areas of expertise: data scientists focus on technical aspects such as data analysis, AI modeling, and algorithms, while clinicians prioritize patient care, medical experience, and clinical judgment. These differing approaches can lead to challenges in working together, as they may not fully comprehend each other's priorities, techniques, strengths, or limitations. Communication challenges arise when data scientists and clinicians struggle to share insights clearly. Data scientists may find it difficult to explain complex algorithms, while clinicians may not convey the nuances of patient care, leading to confusion and difficulties in reaching data-driven healthcare decisions. To bridge these gaps, both groups need to understand each other's roles and work together more effectively. Data scientists can simplify their explanations to make their findings more accessible to clinicians, while clinicians can help data scientists grasp the complexities of patient care. This collaboration ensures that AI and data tools are applied in ways that truly benefit patients (120).
- **Necessary Shifts of Perspective:** For successful collaboration between oncologists and data scientists, a cultural shift is necessary. Oncologists must view data science as a valuable tool to enhance patient care, while data scientists should gain a deeper understanding of clinical challenges. Clear communication and mutual trust are vital, with data scientists simplifying their findings and oncologists embracing data-driven insights. Both groups should actively learn from each other's expertise. This collaboration can lead to more informed decision-making and improved patient outcomes by integrating clinical knowledge with data-driven tools.

### 5.2 Opportunities for overcoming obstacles

#### 5.2.1 Federated learning

Federated learning (FL) is a shared global model training framework that keeps data localized and addresses privacy concerns associated with sensitive and fragmented datasets (84, 121–124). Previously, model training has involved aggregating data from various centers all together, but in FL, each center completes part of the overall operation without its own data being accessible to other centers. Examples of implementation include improving hospital mortality prediction (125) and monitoring air pollution (126). As discussed, many medical and medical research centers in developing countries lack the infrastructure for high-bandwidth data transfers and processes with high computational cost. We see FL as a potential solution to this problem because the local operations conducted require less-intensive data transfers and lower computational costs. Nvidia^14^, a primary leader in the production of AI-capable hardware, has taken a keen interest in FL, which bodes well for LMICs as they partner with Nvidia to increase their computing infrastructure.

#### 5.2.2 Low-cost AI models

Low-cost AI models play a crucial role in addressing the unique challenges faced by LMICs, where resource constraints often limit access to advanced technologies. These models, designed to operate efficiently on low-power devices or with limited computational resources, have demonstrated significant potential across various domains. For instance, Zehra et al. (127) highlight strategies for implementing cost-effective AI-driven digital pathology, enabling earlier and more accessible diagnoses in resource-constrained healthcare systems. Similarly, Gangavarapu (128) introduces a multilingual medical AI model tailored to reduce health disparities in low-resource regions, emphasizing adaptability and equitable access. Beyond healthcare, initiatives like MAIScope (129), a portable AI-powered microscope, showcase how low-cost solutions can automate disease diagnoses in remote settings, bridging critical gaps in healthcare delivery. Divyashree et al. (130) aim to build an affordable healthcare system by integrating low-cost sensors, IoT, and AI to deliver real-time monitoring and diagnostic solutions, making healthcare more accessible and reliable for underserved populations. Such efforts underscore the necessity of designing AI models that not only meet technical requirements but also align with the socioeconomic realities of LMICs. As López et al. (131) argue, integrating these solutions into LMIC ecosystems requires collaboration and innovation to overcome barriers like data quality and infrastructure limitations, paving the way for sustainable AI adoption in underserved regions.

These examples further emphasize how low-cost AI models can address systemic challenges in LMICs. For instance, frugal machine learning (FML) emphasizes hardware, model, and data efficiency, enabling AI to be more accessible and sustainable without compromising performance (132). Similarly, field-programmable gate array (FPGA)-based reconfigurable clusters, as demonstrated by Rupanetti et al. (133), offer scalable and cost-effective solutions for machine learning applications in resource-constrained environments, improving parallel processing capabilities. Wearable devices have shown promise in reducing reliance on expensive ICU equipment, while leveraging AI for adaptive decision-making (130, 134). Such approaches not only enhance accessibility but also bridge gaps in healthcare delivery, ensuring that even under-resourced communities benefit from cutting-edge technology. These initiatives highlight the transformative potential of low-cost AI to improve quality of life, support local infrastructure, and promote equity in technology adoption worldwide.

#### 5.2.3 The role of government, NGOs, and the private sector

To ensure the successful integration of data science in cancer care, coordinated efforts from multiple sectors are required:

- **Government Support**: Governments must play an active role in prioritizing investments in health data systems and infrastructure. Policymakers can create enabling environments by establishing regulations that encourage innovation, ensuring data privacy and security, and providing funding for digital health initiatives (135).
- **NGO Involvement**: Non-governmental organizations (NGOs) can act as intermediaries between healthcare systems and international donors, advocating for increased investment in health technologies. NGOs can also contribute to capacity-building efforts by funding training programs and supporting community engagement (136).
- **Private Sector Engagement**: Private companies, especially those in the technology and healthcare industries, can provide expertise and funding for the integration of AI and data science in oncology. These companies can offer scalable solutions, such as cloud-based data storage and AI platforms, that are more accessible in resource-constrained settings (137).

#### 5.2.4 Opportunities for leapfrogging traditional models of cancer care

Despite the challenges, the Global South has a unique opportunity to leapfrog traditional models of cancer care by adopting AI and data science technologies:

- **Leapfrogging Traditional Infrastructure**: LMICs can bypass the need for costly physical infrastructure by adopting cloud-based solutions and mobile health (mHealth) applications. These technologies allow healthcare providers to access and analyze data remotely, making cancer care more scalable and accessible in underserved regions (34).
- **Real-World Data and AI**: By leveraging real-world data (RWD) collected through mobile health platforms, wearables, and telemedicine, LMICs can build datasets that feed into AI-driven cancer care models. These models can then be used to develop context-specific treatment strategies that reflect the unique challenges and disease patterns in these regions (33, 34).
- **Innovative Diagnostics and Treatment Models**: AI-driven diagnostic tools can improve early detection rates by analyzing imaging and pathology data with high accuracy. Additionally, AI can help personalize treatment plans based on local disease profiles and available resources, enabling more targeted cancer therapies (51).

#### 5.2.5 Fostering growth and embracing change

Adopting data science and artificial intelligence (AI) in healthcare, especially in LMICs, presents exciting opportunities for growth and development. Embracing these new methodologies can help healthcare professionals enhance their work, while also recognizing that change can be challenging. Several theories can guide this transition, supporting healthcare professionals as they explore new approaches and thoughtfully adjust established practices.

- Transformational Learning Theory: Transformational learning, as outlined by Mezirow (138), offers a way to approach change with openness and reflection. In healthcare, this means taking the time to evaluate long-standing methods and consider how data-driven technologies might complement or enhance them. This theory emphasizes that by reflecting on their practices and outcomes, professionals can make thoughtful shifts toward integrating AI and data science. Transformational learning can help healthcare teams embrace these technologies as useful tools in improving patient care and outcomes, especially when applied within tumor boards where personalized, data-driven decisions can have a profound impact.
- **Diffusion of Innovations Theory**: Everett Rogers' Diffusion of Innovations Theory (139), explains how new ideas and technologies are gradually adopted within a community. In the context of healthcare, early adopters of AI and data science—often those who are passionate about innovation—can inspire others to consider these tools. As more professionals witness the positive outcomes that data science can offer, such as improved diagnosis and treatment, the enthusiasm for adopting new methods grows naturally. This collaborative and gradual process allows professionals to learn from each other and integrate AI into their work at a pace that feels comfortable and achievable.
- **Unlearning and Relearning**: In any field, letting go of established ways can feel daunting. However, the concept of unlearning and relearning (140) recognizes that professionals are constantly growing and evolving. In healthcare, this means acknowledging the strengths of traditional methods while remaining open to new possibilities that data science and AI provide. Training programs, workshops, and collaborations can help ease the transition by providing hands-on experiences and support. Rather than viewing this as abandoning old practices, it's an opportunity to build on them by integrating new knowledge that can further enhance patient care.
- **Growth Mindset**: Carol Dweck's Growth Mindset Theory (141) highlights the importance of viewing challenges as opportunities for growth. In healthcare, professionals who approach AI and data science with a growth mindset are likely to see these tools as a way to expand their skills and improve their ability to care for patients. Encouraging a growth mindset across teams fosters a culture where professionals feel supported in learning new techniques, making the adoption of AI and data science a shared journey toward better healthcare.

### 5.3 Proposed capacity-building roadmap

This section outlines a comprehensive capacity-building roadmap aimed at training and integrating data scientists into tumor boards in LMICs. By leveraging academic partnerships, virtual training modules, and regionally aligned certification programs, this framework addresses resource constraints, skills gaps, and infrastructural challenges unique to these settings. It provides a scalable and adaptable pathway for equipping LMIC healthcare systems with the expertise needed to harness data-driven cancer care innovations effectively.

#### 5.3.1 Needs assessment and stakeholder engagement

Capacity-building initiatives must begin with a thorough understanding of the specific challenges faced by LMIC healthcare systems. Surveys and focus groups should be conducted with tumor boards, healthcare providers, academic institutions, and government stakeholders to identify current gaps in skills, resources, and infrastructure. For example, many LMICs lack access to annotated clinical datasets, computational tools, and trained personnel for data-intensive tasks.

Building a consortium of stakeholders, including international academic institutions, regional healthcare organizations, and private sector partners, ensures the initiative is contextually relevant. Engagement with patient advocacy groups further aligns training programs with community-centered healthcare goals.

#### 5.3.2 Foundational framework development

A foundational framework provides the competency benchmarks necessary for effective training and professional development. Drawing on global standards like the EDISON Data Science Competence Framework (43–46), this roadmap emphasizes interdisciplinary knowledge across oncology, data analytics, and machine learning. Specific competencies include tumor biology modeling, predictive analytics, and ethical considerations in data sharing.

Frameworks must also consider the sociocultural and economic contexts of LMICs, ensuring alignment with local healthcare priorities and available resources. Regional collaboration, such as through ASEAN or African Union initiatives, can drive the development of frameworks tailored to shared challenges.

#### 5.3.3 Curriculum and resource development

Curriculum development is pivotal in equipping data scientists with both theoretical and practical skills. Academic partnerships can facilitate the co-creation of open-access curricula, emphasizing modular and flexible learning paths. Virtual training platforms like Coursera or edX, tailored for low-bandwidth environments, can provide asynchronous learning opportunities.

Hands-on training should be prioritized by integrating real-world case studies relevant to tumor board decision-making. Open-source computational tools and healthcare datasets, such as simulated tumor registries, enable practical skill acquisition even in resource-constrained settings.

#### 5.3.4 Pilot programs and infrastructure building

Pilot programs serve as a testing ground for scaling training initiatives. These programs should be launched in select LMIC regions with strong healthcare and academic infrastructure, such as regional centers of excellence. Initial efforts could focus on equipping hospitals with basic computational resources and infrastructure and engaging local experts as trainers and mentors.

Collaborative public-private partnerships can address infrastructure gaps by providing access to cloud computing and storage solutions. Pilot sites must incorporate robust evaluation metrics to refine program delivery and outcomes.

#### 5.3.5 Certification and accreditation

Formal certification ensures that trained data scientists are recognized for their competencies, facilitating their integration into tumor boards. Developing a unified certification framework for specific regions [e.g., ASEAN, South Asian Association for Regional Cooperation (SAARC), African Union] enhances portability and consistency of credentials across borders. The creation of tiered certifications (basic, intermediate, advanced) allows for progressive skill development.

International accreditation bodies, such as ACM or WHO, can partner with regional institutions to standardize training programs. Accredited training centers within LMICs can sustain capacity-building efforts and encourage local leadership in data science education.

#### 5.3.6 Scaling and continuous improvement

Scaling capacity-building efforts requires an iterative approach that incorporates feedback from pilot programs. Successful models can be expanded to additional LMICs, leveraging lessons learned to optimize training methodologies. Continuous engagement with stakeholders ensures curricula remain aligned with evolving healthcare and technological landscapes.

Regular updates to learning materials, such as new case studies or AI tools, maintain the relevance and quality of training programs. Regional and global funding initiatives must be actively pursued to sustain long-term capacity building.

Through this capacity-building roadmap initiative, LMICs can transform their healthcare systems into hubs of data-driven care, realizing the promise of equitable cancer treatment and research worldwide.

## 6 Conclusion

The need for data scientists in tumor boards is growing rapidly across the globe, with AI and machine learning playing an increasingly vital role in cancer care. Data scientists provide the expertise needed to analyze complex datasets, enabling healthcare teams to make data-driven decisions that enhance treatment outcomes. In resource-rich countries, this integration is already proving transformative.

However, the potential impact of data science in the Global South is even more profound. By investing in digital health infrastructure, education, and collaboration, LMICs can leverage AI to improve cancer care in ways that were previously unattainable. Data science offers opportunities to overcome traditional healthcare barriers, enabling more personalized, accessible, and cost-effective cancer treatments.

To achieve this vision, global investment in data science is crucial, particularly in resource-constrained settings. Governments, NGOs, and the private sector must collaborate to support the integration of data scientists into cancer treatment teams, providing the infrastructure, training, and resources needed for success. The future of cancer care in the Global South depends on the capacity to harness the power of data science, and the time to act is now.

## Footnotes

1 See https://nanostring.com/products/geomx-digital-spatial-profiler/geomx-dsp-overview/, Last accessed: January 11, 2025.

2 See https://nanostring.com/products/cosmx-spatial-molecular-imager/, Last accessed: January 11, 2025.

3 See https://www.10xgenomics.com/platforms/visium, Last accessed: January 11, 2025.

4 See https://www.10xgenomics.com/platforms/xenium, Last accessed: January 11, 2025.

5 See https://nanostring.com/products/cosmx-spatial-molecular-imager/single-cell-imaging-overview/, Last accessed: January 11, 2025.

6 See https://nanostring.com/resources/cosmx-smi-vs-xenium-superior-in-situ-single-cell-performance-study/, Last accessed: January 11, 2025.

7 See https://www.10xgenomics.com/products/visium-hd-spatial-gene-expression, Last accessed: January 11, 2025.

8 See https://asc.llnl.gov/exascale/el-capitan, Last accessed: January 11, 2025.

9 See https://www.anl.gov/aurora, Last accessed: January 11, 2025.

10 See https://www.bnamericas.com/en/news/petrobras-latest-supercomputer-goes-fully-operational, Last accessed: January 11, 2025.

11 See https://petrobras.com.br/en/quem-somos/perfil, Last accessed: January 11, 2025.

12 See https://toubkal.um6p.ma/, Last accessed: January 11, 2025.

13 See https://www.nscc.sg/aspire-2a-plus/, Last accessed: January 11, 2025.

14 See https://www.nvidia.com/en-us/, Last accessed: January 11, 2025.
