# Correction: The data scientist as a mainstay of the tumor board: global implications and opportunities for the global south

**DOI:** 10.3389/fdgth.2026.1775230

**Published:** 2026-01-30

**Authors:** Myles Joshua Toledo Tan, Daniel Andrew Lichlyter, Nicholle Mae Amor Tan Maravilla, Weston John Schrock, Frederic Ivan Leong Ting, Joanna Marie Choa-Go, Kishi Kobe Francisco, Mickael Cavanaugh Byers, Hezerul Abdul Karim, Nouar AlDahoul

**Affiliations:** 1 Department of Electrical and Computer Engineering, Herbert Wertheim College of Engineering, University of Florida, Gainesville, FL, United States; 2 Department of Epidemiology, College of Public Health & Health Professions and College of Medicine, University of Florida, Gainesville, FL, United States; 3 Biology Program, College of Arts and Sciences, University of St. La Salle, Bacolod, Philippines; 4 Department of Natural Sciences, College of Arts and Sciences, University of St. La Salle, Bacolod, Philippines; 5 Department of Chemical Engineering, College of Engineering and Technology, University of St. La Salle, Bacolod, Philippines; 6 Department of Electronics Engineering, College of Engineering and Technology, University of St. La Salle, Bacolod, Philippines; 7 Yo-Vivo Corporation, Bacolod, Philippines; 8 College of Medicine, University of Florida, Gainesville, FL, United States; 9 College of Pharmacy, University of Florida, Gainesville, FL, United States; 10 VA North Florida/South Georgia Veterans Health System, Gainesville, FL, United States; 11 Department of Clinical Sciences, College of Medicine, University of St. La Salle, Bacolod, Philippines; 12 Division of Oncology, Department of Internal Medicine, Corazon Locsin Montelibano Memorial Regional Hospital, Bacolod, Philippines; 13 Department of Internal Medicine, Dr. Pablo O. Torre Memorial Hospital, Bacolod, Philippines; 14 Department of Radiology, The Doctors’ Hospital, Inc., Bacolod, Philippines; 15 Department of Diagnostic Imaging and Radiologic Sciences, Corazon Locsin Montelibano Memorial Regional Hospital, Bacolod, Philippines; 16 Department of Civil and Coastal Engineering, Herbert Wertheim College of Engineering, University of Florida, Gainesville, FL, United States; 17 Faculty of Engineering, Multimedia University, Persiaran Multimedia, Cyberjaya, Malaysia; 18 Department of Computer Science, Division of Science, New York University Abu Dhabi, Abu Dhabi, United Arab Emirates

**Keywords:** cancer care, data science, global south, low- and middle-income countries, medical artificial intelligence, personalized medicine, tumor board, transdisciplinarity

The following corrections have been made to the published article:

“Al” was corrected to “AI”. A correction has been made to the section **2 Global perspective: the role of data scientists in tumor boards**, *2.4 Challenges of integrating data science into medical practice on a global scale*, first paragraph:

“AI is a promising innovation in medical imaging, with applications ranging from image acquisition and processing to reporting, follow-up planning, and data management.”

“United States Academy International Development” was corrected to “United States Agency for International Development”. A correction has been made to the section **3 The case for the global south: maximizing the impact of data scientists**, *3.2. Leveraging AI and machine learning: how data scientists can address disparities and improve early diagnosis and treatment*, second paragraph:

“Moreover, the United States Agency for International Development (USAID) has already been making efforts to address the gap by identifying the challenges to large-scale AI implementation in LMICs and highlighting the actions that can most effectively promote the appropriate use of AI to enhance health outcomes in these settings (36).”

The word “foundational” was corrected to “foundation”. A correction has been made to the title of subsection *4.7 Why a data scientist who stays current with foundational models is invaluable*:

“4.7 Why a data scientist who stays current with foundation models is invaluable”

A closing parenthesis was erroneously included after the word “decisions”. A correction has been made to the section **4 Transdisciplinarity for improved patient care: the future of cancer treatment**, *4.7 Why a data scientist who stays current with foundation models is invaluable*, first paragraph:

“Data scientists who are familiar with the latest models—such as GPT, BERT, PaLM, and GNNs—can integrate insights from clinical, genomic, and lifestyle datasets to inform the tumor board's decisions.”

The word “arised” should have read “arisen”. A correction has been made to the section **5 Overcoming barriers to data science integration in the global south**, *5.1 Identifying key barriers, 5.1.4 Risks of over-reliance on AI*, first paragraph:

“Thus, skepticisms may have arisen from limited understanding and data privacy concerns (119).”

The words “Like in” and “, they” were removed, the word “fictional” was inserted before “story”. A correction has been made to the section **5 Overcoming barriers to data science integration in the global south**, *5.1 Identifying key barriers, 5.1.4 Risks of over-reliance on AI*, second paragraph:

“Many medical schools present a fictional story about a woman who visits her doctor to receive her biopsy results.”

The reference for 36 was erroneously written as “United States Academy International Development. Artificial intelligence in global health: defining a collective path forward. United States Academy International Development (2022). Available online at: https://www.usaid.gov/sites/default/files/2022-05/AI-in-Global-Health_webFinal_508.pdf (Accessed on: December 12, 2024”. It should be “United States Agency for International Development. Artificial Intelligence in Global Health: Defining a Collective Path Forward. United States Agency for International Development (2022). Available online at: https://digital.library.unt.edu/ark:/67531/metadc2289561/ (accessed December 12, 2024)”.

The original version of this article has been updated.

